# Reduced Road Injuries While Commuting Due to Heavy Snowfall and Ensuing Modal Shifts Among Junior High School Students in Japan

**DOI:** 10.2188/jea.JE20200504

**Published:** 2022-09-05

**Authors:** Haruhiko Inada, Jun Tomio, Masao Ichikawa, Shinji Nakahara

**Affiliations:** 1 Johns Hopkins Bloomberg School of Public Health, Maryland, United States of America; 2 Graduate School of Medicine, University of Tokyo, Tokyo, Japan; 3 Faculty of Medicine, University of Tsukuba, Ibaraki, Japan; 4 Graduate School of Health Innovation, Kanagawa University of Human Services, Kanagawa, Japan

**Keywords:** epidemiology, injuries, transportation, adolescent

## Abstract

**Background:**

Modal shifts in transport may reduce overall road injuries. Cyclist junior high school students are at a high risk of road injuries while commuting in Japan, and injuries among junior high school students could be reduced if the cyclists switch to other transport modes.

**Methods:**

We estimated the change in the incidence of road deaths and serious injuries while commuting in months with heavy snowfall, when cyclists are likely to switch to other transport modes. Using police data on the monthly number of road injuries while commuting among junior high school students in Japan between 2004 and 2013 and corresponding population statistics and snowfall data, we calculated the monthly injury rate (number of deaths and serious injuries divided by population) at the prefecture level. We conducted Poisson regression analysis to estimate the change in the rate in months with a snowfall of ≥100 cm, compared to months without snowfall.

**Results:**

A total of 3,164 deaths and serious injuries occurred during 2004 to 2013. The injury rate among cyclists was almost zero in months with a snowfall of ≥100 cm. That among cyclists and pedestrians in these months was reduced by 68% (95% confidence interval, 43–82%).

**Conclusion:**

In months with heavy snowfall, road injuries while commuting were reduced due to the near-elimination of cycling injuries among junior high school students in Japan. Switching from cycling to other transport modes would reduce overall road injuries among this population, and inducing modal shifts can be an important tool for road safety.

## INTRODUCTION

Globally, every year, over 1.3 million people die and over 54 million people are injured on roads, and road injury is the leading cause of death among people aged 5–29 years.^1^^,^^2^ To address this enormous burden of road injuries, the United Nations launched the Decade of Action for Road Safety 2011–2020, ‘with a goal to stabilize and then reduce the forecast level of road traffic fatalities around the world’,^3^ which was succeeded by the Second Decade of Action for Road Safety 2021–2030, ‘with a goal of reducing road traffic deaths and injuries by at least 50 per cent’ during the period.^4^ Additionally, it included Target 3.6: ‘By 2020, halve the number of global deaths and injuries from road traffic accidents’ in the Sustainable Development Goals.^5^ However, if current trends continue, it is unlikely that these goals and targets could be achieved.^6^^–^^8^ Therefore, globally, efforts to reduce the burden of road injuries need to be enhanced.

As modal shifts from risky to safer transport modes have a potential to reduce road injuries, such measures have garnered attention recently.^9^^,^^10^ Of all global road deaths, pedestrians, cyclists, motorcyclists, and four-wheeled vehicle occupants comprised 23%, 3%, 28%, and 29% of the fatalities, respectively.^1^ However, there is a wide gap in injury risk among different transport modes.^11^^–^^14^ Regarding the effects of modal shifts on road safety, several studies have reported before-after changes in the number of motor vehicle collisions or road injuries along streets where public transport treatments, such as a bus rapid transit system and bus priority lanes, were introduced, but their results are mixed.^15^^–^^20^ Some other studies have examined the ecological association between area characteristics and the incidence of road injuries within each area. For example, the share of public transport use was inversely associated with the incidence of road injuries in Greater Melbourne, Australia.^21^ Similarly, an increase in public transport use was associated with a decrease in the incidence of road deaths.^10^ However, because of limitations in the study design or population, none of these studies prove that modal shifts reduce road injuries at the regional or national level.

To address this knowledge gap, we analysed changes in road injuries due to spontaneously arising periodical modal shifts while commuting among junior high school students in Japan. Junior high school students commuting by bicycle are at a higher risk of road injuries than are those commuting by walking,^22^ and switching to other, safer transport modes, such as walking, may substantially reduce road injuries among this population. To estimate the proportion of road injuries among students that could be prevented by such modal shifts while commuting, we utilized an innovative approach by leveraging the fact that heavy snowfall makes it impossible for students to commute by bicycle. This, in turn, makes them switch to other transport modes during winter in some regions in Japan. Therefore, the objective of the current study was to estimate the change in the incidence of road injuries while commuting in months with heavy snowfall among junior high school students in Japan. Specifically, we hypothesized that road injuries decrease in months with heavy snowfall.

## METHODS

### Study design

This cross-sectional ecological study aimed to estimate the change in the incidence of road injuries while commuting in months with heavy snowfall and ensuing modal shifts from cycling to other modes among junior high school students in Japan. We quantified the change in the road injury rate among cyclists and pedestrians in months with a snowfall of ≥100 cm, compared to months without snowfall.

### Study settings

In Japan, road injury is a leading cause of mortality and morbidity, and cyclists and pedestrians account for half of all road deaths.^1^ The proportion is even higher among children and adolescents. From 2014 to 2017, 65% of all road injuries among school-age children and adolescents occurred with cyclists and pedestrians.^23^ The main transport modes for school commuting are walking, cycling, and public transport (ie, few students commute by private motor vehicle, except in winter in some areas with heavy snowfall where otherwise commuting is impossible for some students).^22^^,^^24^ Further, a large proportion of road traffic injuries among school-aged children occur during commuting. Specifically, crashes while commuting account for 35% of all pedestrian injuries among elementary school-aged (6–12 years) children and 63% of all cyclist injuries among junior high and high school-aged (12–18 years) adolescents.^23^

### Data sources and variables

Our study population comprised all junior high school students in Japan, aged 12–15 years between 2004 and 2013. From the Institute for Traffic Accident Research and Data Analysis, we obtained national police data on the monthly number of students who died or were seriously injured in a road traffic crash on their way to or from school. To serve as the denominator for the death and serious injury rate (hereafter referred to as the ‘injury rate’), we also obtained population statistics on the annual number of junior high school students from the national school survey.^25^

The road injury data were stratified by year, month, prefecture (47 subnational regions), severity of injury (death or serious injury), sex, and mode of transport (pedestrian or cyclist) of the child. The road injury data were recorded according to a standardized definition of measurements.^26^ Death was defined as those that occurred within 24 hours of the crash, and serious injury was defined as those that were estimated by the physician to require medical care for ≥30 days after the crash.^26^ We excluded injuries of transport modes other than walking and cycling from our study because their number would be negligibly low, as the size of the population at risk of such injuries and their risk of injury would be small. The population statistics data were stratified by year, prefecture, and sex. We assumed that the population size is constant for each school year (from April to March).

To quantify the effect of snowfall on the injury rate, we also obtained data on the monthly amount of snowfall in prefectural capital cities between 2004 and 2013 from the Japan Meteorological Agency.^27^ We used the monthly snowfall in each city as a representative of the entire prefecture. Since the snowfall data were unavailable in the capital cities of Saitama and Shiga Prefectures, we used the data from the city of Kumagaya for Saitama and those from the city of Hikone for Shiga.

### Outcome variable for evaluation

We calculated the injury rate for each year and month, prefecture, and sex. The numerator for the rate was the monthly number of deaths and serious injuries, and the denominator was the corresponding yearly number of students divided by 12 × 100,000; and therefore, the unit of the rate was cases per 100,000 person-years.

### Statistical analysis

To observe the association between the injury rate and amount of snowfall, we plotted the monthly injury rate against the monthly amount of snowfall, stratified by prefecture and mode of transport, in 11 prefectures that had a monthly snowfall of ≥100 cm in at least 1 month between 2004 and 2013 (Akita, Aomori, Fukui, Hokkaido, Ishikawa, Iwate, Nagano, Niigata, Tottori, Toyama, and Yamagata). From the scatterplots, we decided to categorize the monthly amount of snowfall into 0 cm, 1–24 cm, 25–49 cm, 50–99 cm, and ≥100 cm. Then, using the data for all 47 prefectures, we calculated the injury rate stratified by sex, mode of transport, and category of monthly amount of snowfall. In addition, to compare seasonal patterns of the injury rate between the 11 heavy snow prefectures and 12 prefectures that had little snow, which were defined as never having a monthly snowfall of ≥10 cm during the study period (Chiba, Ehime, Hyogo, Kumamoto, Miyazaki, Oita, Okayama, Okinawa, Osaka, Shizuoka, Tokushima, and Wakayama), we also plotted their monthly injury rate averaged between 2004 and 2013, stratified by prefecture and mode of transport.

We estimated the injury rate ratios for each category of monthly amount of snowfall as compared to months without snowfall (ie, 0 cm) using Poisson regression analysis with generalized estimating equations (GEE) to account for clustering within prefecture.^28^ We applied the GEE approach to estimate the marginal association between the amount of snowfall and injury rate. We used the number of deaths and serious injuries and the natural logarithm of the number of junior high school students as the dependent variable and offset term, respectively. We constructed the models separately for four subgroups stratified by sex and mode of transport, two subgroups stratified only by sex, and all cyclists and pedestrians of both sexes. The result of primary interest was the injury rate ratio in months with a snowfall of ≥100 cm for all cyclists and pedestrians of both sexes, which estimated the reduction in the overall injury rate when almost all cyclists switched to other transport modes for commuting in prefectures that had a monthly snowfall of ≥100 cm in at least 1 month between 2004 and 2013, controlling for yearly and monthly patterns and monthly snowfall in all prefectures. To adjust for yearly and monthly patterns of the rate that are independent of the amount of snowfall, including those due to the change in time at risk (ie, students commute less often in some months due to holidays), we input year and month as categorical variables or natural cubic splines with three degrees of freedom with or without their product terms. We considered three within-prefecture correlation structures (independence, exchangeable, and autocorrelated) and selected the best model and structure based on the correlation information criterion.^29^ We examined whether the data were over- or under-dispersed than a Poisson distribution and calculated robust variance estimates.

We conducted all the statistical analyses using R version 4.0.2 (R Foundation for Statistical Computing, Vienna, Austria) and used the geeglm function of the geepack package for GEE and the ns function of the splines package for natural splines.^30^^,^^31^ This study did not require an institutional review board approval because it is an observational study that used only aggregate public domain data.

## RESULTS

**Figure 1.**
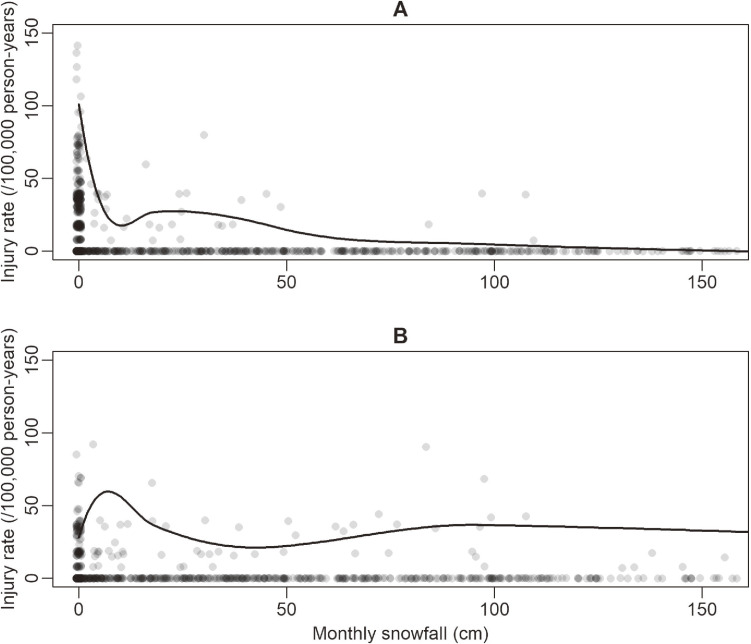
Monthly injury rates among junior high school students while commuting and snowfall in 11 prefectures^a^ in Japan in 2004–2013 for (**A**) cyclists and (**B**) pedestrians. The solid lines are robust locally weighted regression smoothers exaggerated by 10 times for visibility.^40^ The figure does not show 38 prefecture-months with a monthly snowfall of greater than 160 cm, in which there were no deaths or serious injuries among cyclists and seven deaths and serious injuries among pedestrians. ^a^These prefectures had a monthly snowfall of ≥100 cm in their capital city in at least one month between 2004 and 2013: Akita, Aomori, Fukui, Hokkaido, Ishikawa, Iwate, Nagano, Niigata, Tottori, Toyama, and Yamagata.

Between 2004 and 2013, 3,164 deaths and serious injuries (844 female cyclists, 1,516 male cyclists, 371 female pedestrians, and 433 male pedestrians) occurred among junior high school students while commuting in Japan. Figure 1 shows the association between monthly injury rates and monthly amount of snowfall for cyclists and pedestrians separately in the 11 prefectures that experienced a monthly snowfall of ≥100 cm in at least 1 month between 2004 and 2013. Among cyclists, the injury rate showed a sharp decline with the increase in snowfall and it was almost zero in months with a snowfall of ≥100 cm (Figure 1A). Among pedestrians, no specific trend was observed between the injury rate and amount of snowfall (Figure 1B).

**Figure 2.**
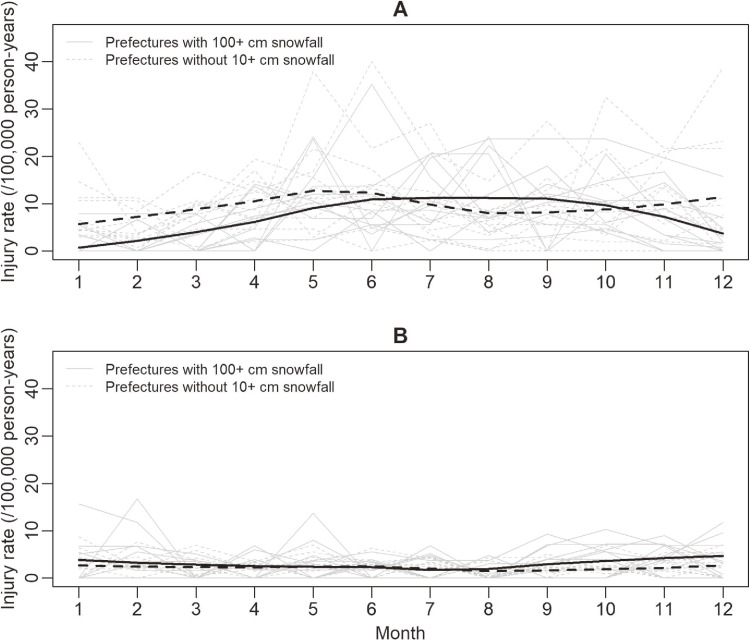
Seasonal patterns of injury rates among junior high school students while commuting in 11 prefectures with heavy snow^a^ and 12 prefectures with little snow^b^ in Japan in 2004–2013 for (**A**) cyclists and (**B**) pedestrians. The black lines are robust locally weighted regression smoothers.^40^ ^a^These prefectures had a monthly snowfall of ≥100 cm in their capital city in at least one month between 2004 and 2013: Akita, Aomori, Fukui, Hokkaido, Ishikawa, Iwate, Nagano, Niigata, Tottori, Toyama, and Yamagata. ^b^These prefectures never had a monthly snowfall of ≥10 cm in their capital city between 2004 and 2013: Chiba, Ehime, Hyogo, Kumamoto, Miyazaki, Oita, Okayama, Okinawa, Osaka, Shizuoka, Tokushima, and Wakayama.

Figure 2 shows seasonal patterns of injury rates in the 11 prefectures with heavy snow and the 12 prefectures with little snow. The injury rate among cyclists in the 11 prefectures with heavy snow showed a unimodal pattern: low in winter months (December through February) and high in late spring, summer, and fall months (Figure 2A). On the other hand, the injury rate among cyclists in the 12 prefectures with little snow was relatively flat with small peaks in May and December (Figure 2A). The injury rate among pedestrians was higher in the 11 snowy prefectures than the 12 snowless prefectures in late fall and winter months (Figure 2B). The injury rates among both cyclists and pedestrians were similar between the two groups of prefectures in May through October (Figure 2A and Figure 2B).

Table 1 presents the association between monthly injury rates and category of monthly amount of snowfall stratified by sex and mode of transport, using data from all prefectures. It shows similar associations between injury rates and amount of snowfall compared to those presented in Figure 1. Specifically, with an increase in snowfall, a sharp decline was observed in the injury rate among cyclists, and no specific trend or possibly an inverse-U trend was observed among pedestrians. The steep decline was more prominent among female cyclists as compared to their male peers in months with a snowfall of <100 cm.

**Table 1.** Monthly amount of snowfall and the injury rates^a^ by sex and mode of transport

Sex	Monthly snowfall^b^	Cyclists and pedestrians^c^		Cyclists^c^		Pedestrians^c^		Number of prefecture-months
Female	0 cm	7.3	(1,107)	5.2	(797)	2.0	(310)	4,725
	1–24 cm	4.8	(72)	2.6	(38)	2.3	(34)	509
	25–49 cm	4.5	(14)	2.3	(7)	2.3	(7)	149
	50–99 cm	5.2	(15)	0.4	(1)	4.8	(14)	146
	≥100 cm	2.4	(7)	0.3	(1)	2.0	(6)	111^d^

	Total	6.9	(1,215)	4.8	(844)	2.1	(371)	5,640

Male	0 cm	10.9	(1,732)	8.6	(1,378)	2.2	(354)	4,725
	1–24 cm	10.5	(164)	7.3	(114)	3.2	(50)	509
	25–49 cm	8.7	(28)	5.6	(18)	3.1	(10)	149
	50–99 cm	5.3	(16)	1.7	(5)	3.7	(11)	146
	≥100 cm	3.0	(9)	0.3	(1)	2.6	(8)	111^d^

	Total	10.6	(1,949)	8.2	(1,516)	2.3	(433)	5,640

^a^The numerator for the rate was the number of junior high school students who died within 24 hours of the crash or seriously injured and were estimated to require medical treatment for 30 days or more after the crash by the physician in Japan between 2004 and 2013, and the denominator was the sum of the corresponding number of students in each year and prefecture.

^b^The monthly snowfall data in the capital cities of 45 prefectures between 2004 and 2013 were obtained from the Japan Meteorological Agency.^27^ For Saitama and Shiga, the data from another city in the same prefecture were used because of the unavailability of data in their capital cities.

^c^The figures are the injury rate per 100,000 person-years, and the numbers of deaths and serious injuries are in parentheses.

^d^11 prefectures (Akita, Aomori, Fukui, Hokkaido, Ishikawa, Iwate, Nagano, Niigata, Tottori, Toyama, and Yamagata) had at least 1 month with a snowfall of ≥100 cm between 2004 and 2013.

The mean and variance of the data were close to each other, which suggests that they follow a Poisson distribution (data not shown). Table 2 shows the results of the Poisson regression analyses. For all the six subgroups and all cyclists and pedestrians of both sexes, the selected model had year and month as their natural cubic splines without their product terms and an exchangeable correlation structure. The injury rate ratios of cyclists in months with a snowfall of ≥100 cm were almost zero. The injury rate ratios of cyclists and pedestrians combined were similar between the sexes, and that in months with a snowfall of ≥100 cm was 0.32 (95% confidence interval, 0.18–0.57) for cyclists and pedestrians of both sexes.

**Table 2.** Monthly snowfall and the injury rate^a^ ratios by sex and mode of transport

Sex	Monthly snowfall^b^	Cyclists and pedestrians	Cyclists	Pedestrians

		Point estimate^c^ [95% CI] of injury rate ratio	Point estimate^c^ [95% CI] of injury rate ratio	Point estimate^c^ [95% CI] of injury rate ratio
Female	0 cm	(Referent)	(Referent)	(Referent)
	1–24 cm	0.62 [0.48, 0.79]	0.51 [0.37, 0.71]	0.89 [0.55, 1.44]
	25–49 cm	0.46 [0.21, 1.04]	0.44 [0.21, 0.94]	0.64 [0.20, 2.05]
	50–99 cm	0.57 [0.30, 1.09]	0.08 [0.01, 0.50]	1.55 [0.70, 3.45]
	≥100 cm	0.29 [0.13, 0.66]	0.14 [0.06, 0.31]	0.58 [0.19, 1.77]

Male	0 cm	(Referent)	(Referent)	(Referent)
	1–24 cm	1.03 [0.85, 1.26]	0.95 [0.75, 1.21]	1.33 [0.98, 1.82]
	25–49 cm	0.65 [0.42, 1.00]	0.55 [0.31, 1.00]	1.06 [0.48, 2.35]
	50–99 cm	0.45 [0.21, 0.97]	0.18 [0.04, 0.84]	1.30 [0.63, 2.70]
	≥100 cm	0.33 [0.13, 0.83]	0.12 [0.02, 0.60]	0.80 [0.42, 1.50]

Both sexes	0 cm	(Referent)		
	1–24 cm	0.86 [0.72, 1.02]		
	25–49 cm	0.56 [0.36, 0.88]		
	50–99 cm	0.50 [0.28, 0.89]		
	≥100 cm	0.32 [0.18, 0.57]		

CI, confidence interval.

^a^The numerator for the rate was the number of junior high school students who died within 24 hours of the crash or seriously injured and were estimated to require medical treatment for 30 days or more after the crash by the physician in Japan between 2004 and 2013, and the denominator was the sum of the corresponding number of students in each year and prefecture divided by 12.

^b^We obtained the monthly snowfall data in the capital cities of 45 prefectures between 2004 and 2013 from the Japan Meteorological Agency.^27^ For the prefectures of Saitama and Shiga, we used the data from another city in the same prefecture because of the unavailability of data in their capital cities.

^c^We estimated these ratios with Poisson regression analyses with the generalized estimating equations approach,^28^ controlling for yearly and monthly patterns and monthly snowfall in all prefectures. The best models were selected using the correlation information criterion.^29^

## DISCUSSION

### Principal findings

With a monthly snowfall of ≥100 cm, the injury rate was close to zero among cyclists, and the overall injury rate while commuting was reduced by 68%. This is the estimated marginal effect of heavy snowfall on the injury rate for months without snowfall in the 11 prefectures that had a monthly snowfall of ≥100 cm in at least 1 month between 2004 and 2013, controlling for yearly and monthly patterns, and monthly snowfall in all prefectures. This result suggests that modal shifts presumably induced by heavy snowfall, especially from cycling to other modes, would substantially reduce road injuries while commuting among junior high school students in Japan.

### Strengths and weaknesses

To our knowledge, this is the first study that showed that modal shifts could reduce road injuries at the regional level. We did so by leveraging the amount of snowfall, which causes major modal shifts, as the exposure variable. Another strength is the use of national 10-year data of a country with over 120 million individuals, which enabled us to obtain a large sample size and precise estimates. Besides, underreporting of road injuries among pedestrian and cyclist children is small in Japan.^32^

There are multiple sources of bias for the estimated effect size, but it is probably close to what we aimed to estimate; namely, the expected reduction in the injury rate when cyclist junior high school students switch to other transport modes in the whole country, all year round. On one hand, the effect size could have been overestimated if some pedestrians had also switched to other, safer transport modes; or if subgroups of cyclists and pedestrians had decreased injury rates due to reduced traffic volume and speed in months with heavy snowfall. Anecdotally, some schools operate their school bus or allow parents to send their children by their personal car only in winter; however, these arrangements minimally influence pedestrians as shown in the present study (small changes among pedestrians). Snowfalls usually increase risk of non-fatal road traffic injuries rather than reducing them.^33^

On the other hand, the effect size could have been underestimated if the snowfall data we used did not represent the entire prefecture. We used the amount of snowfall in each prefectural capital city. If areas outside the capital city in a prefecture had a different amount of snowfall, this would have biased our results toward the null. Besides, the increased risk of non-fatal road injuries due to snowfall,^33^ also lead to underestimation. These possible biases would have conservative results.

Another reason why we consider the bias to be small is that the estimated effect size of the current study (68% reduction) is similar to an estimation assuming that the injury rate among cyclists (42 per 100,000 person-years) had reduced to that among pedestrians (2.9).^22^ The number of deaths and serious injuries during the study period among cyclists (2,360) would reduce to $2360\times \frac{2.9}{42}=163$ , and the total reduction, if there is no change of the number of deaths and serious injuries among pedestrians (804), would be $(1-\frac{804+163}{804+2360})\times 100=69\%$ .

One of the limitations of our study is that we were unable to use data on the distribution of transport modes for commuting among our study population when there was heavy snowfall. Cyclists are likely to have switched to walking, bus, train, or private motor vehicle. In the 11 snowy prefectures in winter months, the injury rate showed a decrease among cyclists and an increase in among pedestrians, which supports this hypothesis (Figure 2A and Figure 2B). Probably many students switched to walking because over 83% of them live within 2 km of their school,^34^ which would add health benefits by increasing their physical activity. However, some cyclists and pedestrians might have switched to a private motor vehicle. Commuting by private motor vehicle could be problematic because evidence shows that active school transport increases children’s physical activity,^35^ and also because it increases parents’ burden. Nevertheless, our findings would inform policymakers, schools, and parents by showing that modal shifts from cycling to other modes can substantially reduce road injuries while commuting. Switching to walking would be suitable for many students, and other options could be school bus, other public transport, and private motor vehicle.

### Strengths and weaknesses in relation to other studies

By utilizing an innovative approach that leveraged the amount of snowfall, for the first time, we showed that modal shifts could dramatically reduce overall road injuries at the regional level. Compared with other studies, our study controlled for the amount of risk exposure among the study population more precisely. As we restricted to road injuries while commuting among junior high school students, their exposure to traffic in terms of the time of the day and the distance was constant across the months within the year, which were the units of our analyses. However, our study population, junior high school students, was demographically and socially narrower in scope than that used in other studies, whose study populations were road users of all ages.^10^^,^^15^^–^^19^^,^^21^

### Meaning of the study

Our findings have important implications for road safety beyond junior high school students in Japan, because they suggest that inducing modal shifts could be an important tool for increasing road safety. While safety measures for cyclists, such as increasing helmet use, redesigning cycle routes so that cyclists are separated from motor vehicles, and area-wide traffic calming, are also important to reduce injuries,^36^^–^^38^ their effects would be smaller or it can be more time-consuming and expensive to implement them.

The generalizability of our findings would depend on the distribution of transport modes for commuting and the risk of road injury while commuting by each transport mode. In countries where cyclists are at a high risk of road injury and where other, safer transport modes are available, switching from cycling to other modes may reduce road injuries while commuting. On the other hand, in areas where few students commute by bicycle, such a reduction would not be observed.

### Unanswered questions and future research

Modal shifts from cycling to other modes entail health consequences via the change in the level of exposure to risk factors other than road traffic, such as physical activity and air pollutants,^39^ and therefore, a health impact assessment that also considers these effects is warranted.

### Conclusion

With a monthly snowfall of ≥100 cm, the injury rate among junior high school students while commuting to and from school decreased by 68%, probably due to modal shifts, such as from cycling to other, safer modes. In Japan, commuting by bicycle is a major cause of road injuries among junior high school students, and policies that limit bicycle use to students who do not have any other means of transportation for commuting should be considered with due respect to a potential change in their level of physical activity and acceptability by them and their parents. Moreover, our findings indicate that inducing modal shifts can be an important tool for increasing road safety.
